# Echoes of Trauma: DSA-Guided Coil Embolization for Carotid Cavernous Fistula Presenting with Ocular Bruit and Pulsatile Exophthalmos

**DOI:** 10.15190/d.2025.2

**Published:** 2025-03-31

**Authors:** Erekle Ekvtimishvil, Arpita Meher, Jessen Johnson

**Affiliations:** ^1^Endovascular Neurosurgery Department, High Technology Medical Center, University Clinic, Tbilisi, Georgia; ^2^Department of Medicine, Tbilisi State Medical University, Tbilisi, Georgia

**Keywords:** Carotid-Cavernous Fistulas, Chemosis, Digital Subtraction Angiography (DSA), Arteriovenous Fistula.

## Abstract

Carotid-Cavernous Fistulas (CCFs) are abnormal arteriovenous connections between the carotid artery and the cavernous sinus, resulting from trauma. This case report presents a 47-year-old male who developed a CCF following a road traffic accident. The patient exhibited symptoms such as pulsatile exophthalmos and an ocular bruit, characteristic of CCFs. Diagnosis was confirmed through Digital Subtraction Angiography (DSA), revealed the origin of the fistula. The patient was treated using a novel combined approach of microsurgery and endovascular intervention, involving ligation of the internal carotid artery and transarterial coil embolization. The treatment resulted in rapid resolution of symptoms, including the pulsatile exophthalmos and ocular bruit. This case highlights the importance of a multidisciplinary approach, blending microsurgical and advanced endovascular techniques, in effectively managing traumatic CCFs. The study underscores the value of early diagnosis and the evolving role of minimally invasive procedures in improving patient outcomes.

## INTRODUCTION

Carotid Cavernous Fistulas (CCFs) are classified based on their origin and flow characteristics. Whether spontaneous or trauma-induced, they often manifest with distinct clinical features such as pulsatile exophthalmos and ocular bruit. Imaging modalities play a crucial role in diagnosis of fistula; therapy is determined by its nature and severity^1-3^. This case demonstrates the effective combination of microsurgical and endovascular procedures, providing valuable insights into the management of CCF. 

This case report describes the presentation, diagnosis, and effective treatment of a 47-year-old Caucasian male. He was diagnosed with CCF with CTA. The patient underwent a combined microsurgical and endovascular treatment. Leading to the successful closure of the fistula and spontaneous recovery.

## CASE PRESENTATION

A 47-year-old Caucasian male presented to the Emergency Room at High Technology Medical Center University Clinic, following a road traffic accident, he reported a severe headache and throbbing eye pain, occasionally affecting his visual clarity.

Upon thorough examination, the patient exhibited chemosis, pulsatile exophthalmos, and an ocular bruit, the classical symptomatic triad indicative of Carotid-Cavernous Fistulas (CCFs). Based on Barrow classification it is Type A (refer to Table 1).

**Table 1:** Table 1. Barrow Classification of Carotid-Cavernous Fistulas (CCFs)^3^.

Types	Description
A	Direct connection between the Internal Carotid Artery and Cavernous Sinus
B	The connection between the dural branches of the Internal Carotid Artery and Cavernous Sinus
C	The connection between dural branches of the External Carotid Artery and Cavernous Sinus
D	The connection between the dural branches of both the Internal Carotid Artery and External Carotid Artery and the Cavernous Sinus

**Figure 1:**
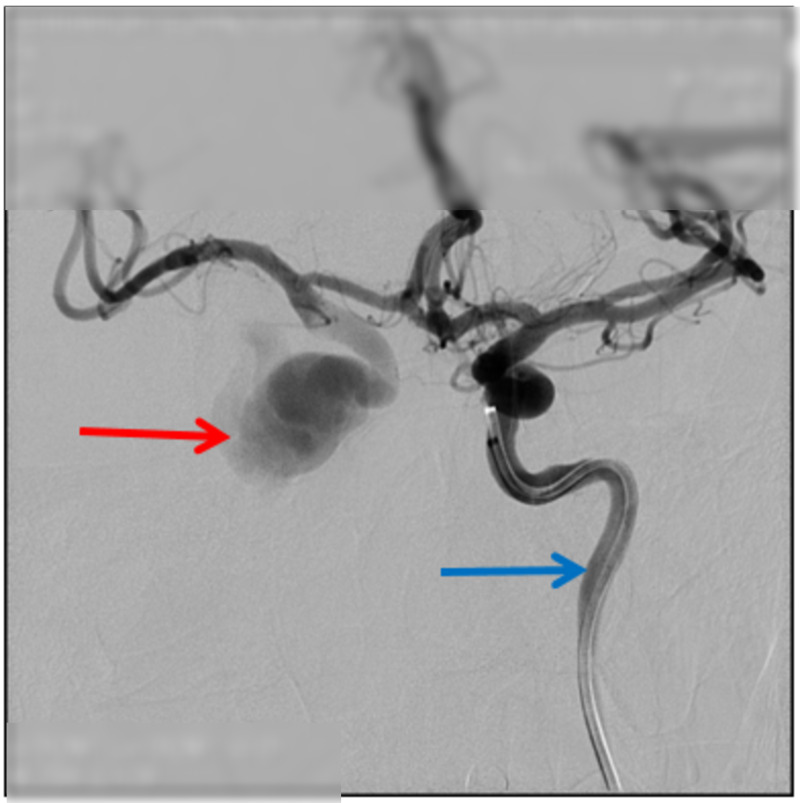
Figure 1. View of conventional DSA of the Left Internal Carotid Artery (blue arrow) The Carotid-Cavernous Fistula (red arrow) of the Right Internal carotid artery and the Cavernous sinus can be identified.

**Figure 2:**
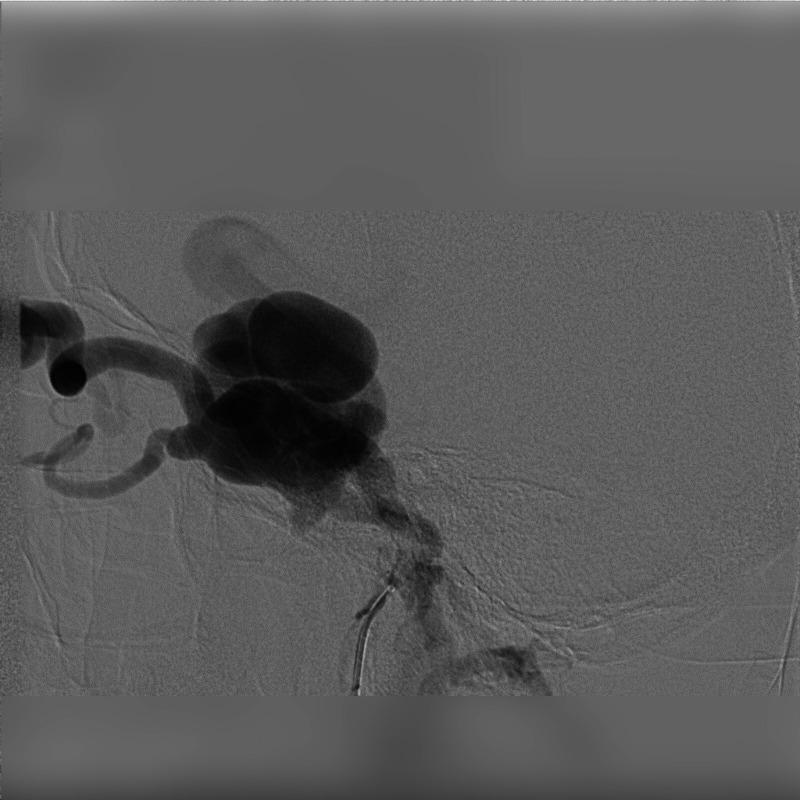
Figure 2. View of DSA showing the CCF draining into the Superior and Inferior Ophthalmic Vein

Subsequent cerebral angiography confirmed the presence of a CCF originating from the Right Internal Carotid Artery, accompanied by multiple traumatic injuries to the cavernous sinus. In the conventional Digital Subtraction Angiography (DSA) image (see Figure 1, the Left Internal Carotid Artery (marked with a blue arrow) is visible, while the carotid-cavernous fistula (marked with a red arrow) involving the right internal carotid artery and the cavernous sinus is also identifiable. In the second DSA view (see Figure 2), the Carotid-Cavernous Fistula (CCF) is observed to drain into both the superior and inferior ophthalmic veins, indicating complex vascular involvement and potential clinical implications.

**Figure 3:**
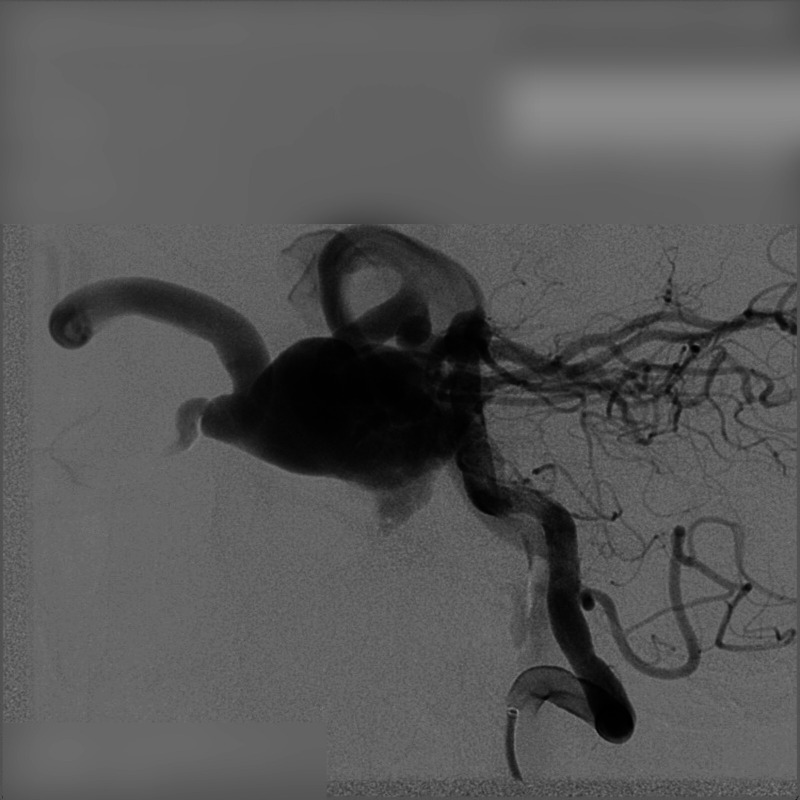
Figure 3. View of conventional DSA of the vertebral basilar system indicating the CCF

In the conventional DSA below (see Figure 3) of the vertebral basilar system, the presence of the CCF is evident, highlighting the need for detailed assessment and careful management of vascular abnormalities in this region.

**Figure 4:**
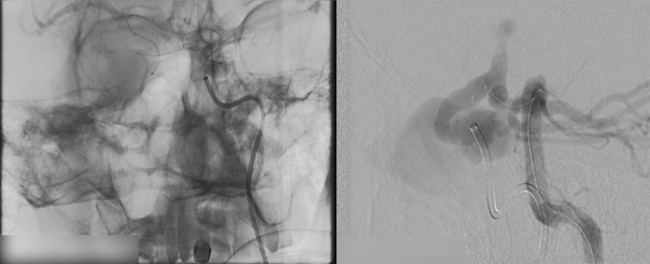
Figure 4. Ligation of the Right internal carotid artery and the microcatheterization through the Anterior Communicating artery and Posterior Communicating artery to introduce coils into the fistula

The CCF was treated using a combination of microsurgical and endovascular procedures. The microsurgical approach involved ligation of the ICA using a standard surgical approach, which interrupted the aberrant blood flow caused by the fistula. The endovascular component used a transarterial technique, with dual microcatheterization into the anterior communicating artery and posterior communicating artery. During this endovascular phase, coils were precisely inserted to occlude and block the aberrant arteriovenous connection, ensuring a comprehensive and successful therapy for CCF. As demonstrated in (see Figure 4), the procedure involved ligation of the right internal carotid artery and microcatheterization through both the anterior communicating and posterior communicating arteries to introduce coils into the fistula, effectively occluding the abnormal vascular connection and restoring normal blood flow dynamics.

**Figure 5:**
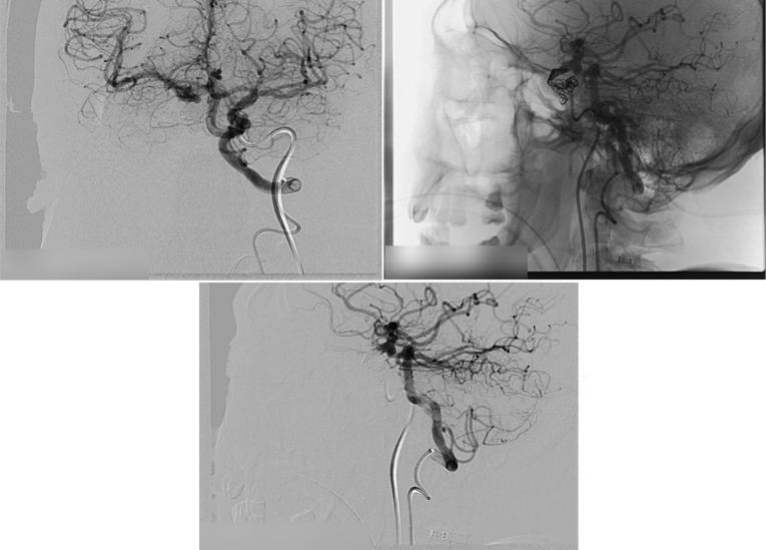
Figure 5. After transarterial coil embolization, the total filling of the Right Hemisphere can be noted from the Left Internal Carotid artery and the vertebral basilar system

Following the procedure, the patient's pulsatile exophthalmos and ocular bruit resolved rapidly within a few hours, while subsequent follow-up revealed a gradual yet consistent improvement in the patient's chemosis. The images below (see Figure 5), depicts transarterial coil embolization, a comprehensive filling of the right hemisphere is observed, facilitated by blood flow redirection from both the left internal carotid artery and the vertebral basilar system, the findings indicate successful closure of the fistula and restoration of cerebral perfusion dynamics.

## DISCUSSION

Arteriovenous abnormalities between the carotid artery or its branches and the cavernous sinus are referred to as carotid-cavernous fistulas (CCFs). These fistulas are typically categorized as direct or indirect, high or low flow, and classified into Barrow types A, B, C, or D^1^. Trauma and spontaneous development are two primary causes of these lesions^2^.

### Classification

 There are two types of carotid-cavernous (CCFs): are direct fistulas (Barrow type A) and dural or indirect fistulas (Barrow types B, C, and D), shown in Table 1^3^.

Indirect fistulas typically exhibit low flow, while direct fistulas are characterized high flow. Type D is the most common form of spontaneous dural carotid-cavernous fistulas. The artery of the inferior cavernous sinus is the most commonly involved trunk of the internal carotid artery (ICA), although dural fistulas can also involve the meningohypophyseal trunk and its branches. The internal maxillary artery is the most commonly involved branch of the external carotid artery, with other implicated branches including the middle and accessory meningeal arteries, ascending pharyngeal artery, anterior deep temporal artery, and posterior auricular artery^3^.

### Aetiology

Several factors can lead to direct fistulas, that are defined by abnormal connections between veins and arteries. These include forceful or penetrating trauma, such as concussions, that can damage normal vascular structures. Direct fistulas can also develop as a result of the rupture of an intracranial aneurysm within the cavernous sinus, a sophisticated network of veins near the base of the brain. Certain genetic disorders, such as connective tissue disease Ehlers-Danlos syndrome type IV, make people more likely to develop direct fistulas. Furthermore, aberrant arteriovenous connections can unintentionally form as a result of iatrogenic interventions such as internal carotid endarterectomy, transarterial endovascular procedures, and surgeries like craniofacial or trans-sphenoidal excision of pituitary tumors^3^.

In contrast, indirect or dural fistulas often have distinct underlying causes. Among these is hypertension, a condition marked by elevated blood pressure, that may weaken blood vessel leading to the development of fistulas. Dural fistulas have also been linked to internal carotid artery (ICA) dissection, a condition in which there is a rupture in the inner lining of the carotid artery, and fibromuscular dysplasia, a disorder that affects the walls of arteries. Interestingly, direct and indirect fistulas are linked to Ehlers-Danlos syndrome type IV, that is characterized by abnormalities in connective tissue. Postmenopausal women, in particular, may be more susceptible to these conditions, likely due to age-related vascular changes and hormonal fluctuations that increase their vulnerability^3^.

### Pathophysiology

The thrombosis of cavernous sinus venous outflow channels, followed by vascular changes that facilitate collateral flow, is believed to be the initial cause of the pathophysiology of CCFs. This theory is widely accepted, as it explains the formation of arteriovenous fistulas affecting various dural sinuses. However, some authors propose an alternative theory, suggesting that the dilation of pre-existing dural-arterial anastomoses leads to the development of CCFs following the rupture of one or more thin-walled dural arteries. The collateral blood supply provided by these anastomoses results in an angiographic appearance similar to that of a congenital vascular abnormality ^3^.

### Symptoms

Carotid-cavernous fistulas (CCFs) are characterized by a range of symptoms indicative of an abnormal connection between the carotid artery and the cavernous sinus, a region housing vital blood vessels and nerves. Patients often report subjective bruits—abnormal sounds generated inside the skull due to turbulent blood flow. Altered vascular dynamics affecting the nerves responsible for eye movement may lead to diplopia (double vision). Patients may also experience eye redness and excessive tearing, which are signs of ocular congestion caused by irregular blood flow. Increased pressure on ocular tissues can result in visual impairment and the sensation of a foreign body in the eye. Headaches are commonly reported, likely due to the vascular alterations and associated pressure effects^3,4^.

Ocular symptoms are more prevalent in anterior draining fistulas. Patients suffering from posteriorly draining fistulas may experience neurologic symptoms such as disorientation and expressive aphasia, as well as diplopia from isolated ocular motor nerve pareses. Clinical symptoms are often more pronounced in direct fistulas, while they tend to be more insidious in cases of direct fistula ^2,3^.

The clinical manifestations of CCFs differ based on the lesion's flow dynamics, specifically on high or low flow. Proptosis, an abnormal protrusion of the eyeball, is a typical sign. In high-flow lesions, proptosis may exhibit a pulsing pattern due to turbulent and rapid blood flow through the abnormal connection between the carotid artery and the cavernous sinus. The conjunctival and episcleral arteries show arterialization, suggesting a change in blood flow dynamics, and the affected eye frequently appears red. Chemosis, or conjunctival swelling, is a common observation. Strabismus, may result from orbital congestion, ocular motor nerve dysfunction, or a combination of both. An ocular bruit— caused by turbulent blood flow may be audible upon inspection. Vascular congestion in the eye can lead to stasis retinopathy and elevated intraocular pressure (IOP). In severe cases, a noticeably raised episcleral venous pressure may lead to the development of central retinal vein blockage. Due to the aberrant vascular dynamics linked to carotid-cavernous fistulas (CCF), acute trauma or ischemia may cause optic neuropathy, which can present as glaucomatous or non-glaucomatous. Taken together, these clinical indicators emphasize the complex neurological and ocular symptoms of CCF, highlighting the significance of early detection and treatment^3,4^.

### Diagnosis

If a CCF is suspected, the assessment may include color doppler imaging, routine tonometry, pneumotonometry, and ultrasonography. Color Doppler is used to detect the direction and velocity of flow in orbital veins helping to determine arterial flow in cases with CCF. For individuals in whom a carotid-cavernous fistula is suspected, neuroimaging is required. This may use non-invasive CTA or MRA. Both methods are highly sensitive in detecting CCFs, especially when visual disturbances are present, regardless of whether the fistulas are diffuse or direct. In certain cases, Digital Subtraction Angiography (DSA) may still be necessary. DSA remains the gold standard for diagnosing and classifying carotid-cavernous fistulas, and it can serve both diagnostic and therapeutic roles. DSA also provides detailed information about the drainage pattern of the fistula, including whether it drains posteriorly via the inferior petrosal sinus (IPS), anteriorly via the superior ophthalmic vein, or both. Additionally, DSA can detect cerebral venous reflux^3-6^.

### Clinical Implications

Carotid-cavernous fistulas (CCFs) present with a variety of symptoms and causes, requiring different treatment approaches. One case involved a 55-year-old female of Chinese origin who developed CCF secondary to a ruptured persistent primitive trigeminal artery (PPTA) aneurysm. She underwent successful endovascular embolization with detachable coils and an Onyx-18 liquid embolic system, which effectively obliterated both the fistula and the aneurysm. Symptoms such as orbital bruit and chemosis resolved immediately post-procedure, and mild residual abducens nerve paresis resolved within a month. This case demonstrates the efficacy of endovascular embolization in treating complex vascular pathologies without significant neurological sequelae^7^.

CCFs can also present atypically, such as with cerebral infarction in the absence of ocular manifestations. One case involved a patient with internal carotid artery (ICA) stenosis who suffered a massive cerebral infarction due to interruption of the ICA blood supply by the CCF. Another case involved traumatic eye rupture leading to CCF and subsequent cerebral infarction. Digital subtraction angiography (DSA) remains the gold standard for diagnosis. While some patients with mild symptoms may benefit from ICA compression, most require endovascular treatment with embolic materials like detachable balloons, laminated stents, spring rings, or Onyx^8^.

In cases of traumatic CCF, a patient with a gunshot wound to the posterior pharynx experienced complex midface fractures and cervical spine injury. Initial imaging revealed vascular trauma, and DSA confirmed CCF with rapid arteriovenous shunting. Due to difficulties accessing the fistula, the decision was made to ligate the internal carotid artery and perform endovascular coiling, successfully resolving the fistula while preserving cerebral perfusion through collateral circulation^9^.

These cases underscore the diverse causes and manifestations of CCF, highlighting the importance of DSA for diagnosis. Endovascular embolization remains the primary treatment, though in challenging cases, ICA ligation may be necessary. Prompt, tailored treatment strategies are essential for optimal outcomes and the prevention of long-term complications.

### Treatment 

The type of treatment is determined by the kind of CCF under investigation, clinical history, and other factors. Most low-flow or Barrow types B, C, or D arteriovenous fistulas resolve on their own and do not require treatment; other approaches may require ongoing observation. The progression of a patient’s symptoms is typically slower with low-flow CCFs. However, because of the relatively high risk of symptom worsening and the potential for brain hemorrhage^1,2^.

For direct CCFs, treatment options were historically limited to either leaving the fistula untreated by ligating the cervical ICA proximal to the fistula and the intracranial ICA distal to it, or opting for observation, or occluding the common carotid artery or intracranial artery -both of which could potentially result in cerebral ischemia due to an embolic event. With the advent of endovascular interventional procedures, open surgical therapies are no longer the preferred approach. Endovascular treatment offers a lower risk of cerebral infarction and is less invasive than ICA sacrifice. The optimal treatment plan is influenced by factors such as the arterial supply, venous drainage, blood flow rate through the fistula, and the patency of the Willis circle. In situations where there are significant ICA wall tears, which could allow the injected embolic material to return to the arterial circulation and put the patient at risk for embolic consequences, flow-diverting stent support may be used for endoluminal repair. These stents can be placed across the ICA tear to prevent the backflow of the injected material^3^.

Treatment options for dural CCFs include observation, IOP-lowering drugs, and stereotactic radiosurgery, intermittent compression of the ipsilateral internal carotid artery or superior ophthalmic vein, and endovascular intervention. If surgical surgery is not required, patients may utilize occlusion methods, such as external manual carotid compression, to facilitate the resolution of the CCF. While a careful waiting approach is appropriate for many individuals with dural CCF, treatment may occasionally be required to prevent long-term effects. Indications for intervention include uncontrollably high IOP, persistent diplopia, and severe proptosis with corneal exposure, optic neuropathy, retinal ischemia, severe bruit, and cerebral venous drainage from the fistula. Endovascular therapy, which can be delivered intravenously or transarterially, is considered the first-line treatment ^3,4^.

- Successful closure of the CCF resulted in the restoration of normal cerebral perfusion dynamics.
- A multidisciplinary treatment approach combining microsurgical and endovascular techniques is required for the effectiveness of the treatment.
- The rapid resolution of symptoms, such as pulsatile exophthalmos and ocular bruit, along with gradual improvement in chemosis, underscores the importance of regular clinical and radiological surveillance to ensure optimal patient outcomes.

## CONCLUSION

This case report underscores the critical importance of a multidisciplinary approach in managing complex traumatic carotid-cavernous fistulas (CCFs). By strategically combining microsurgical ligation of the internal carotid artery with precise endovascular coil embolization, we achieved rapid and significant symptom resolution in a patient with a severe CCF. This combined strategy not only effectively closed the abnormal arteriovenous connection but also restored normal cerebral blood flow, as evidenced by post-procedure imaging. The patient's swift recovery, marked by the elimination of pulsatile exophthalmos, ocular bruit, and progressive improvement in chemosis, highlights the efficacy of this novel treatment paradigm. This case reinforces the value of early and accurate diagnosis through Digital Subtraction Angiography (DSA) and demonstrates the evolving role of minimally invasive techniques in optimizing patient outcomes.

Moreover, it emphasizes the necessity of ongoing clinical and radiological monitoring to ensure sustained recovery and prevent potential complications.

## References

[1] Henderson A D, Miller N R (2017). Carotid-cavernous fistula: current concepts in aetiology, investigation, and management. Eye.

[2] Sumdani Hasan, Aguilar-Salinas Pedro, Avila Mauricio J., El-Ghanem Mohammad, Dumont Travis M. (2021). Carotid Cavernous Fistula Treatment via Flow Diversion: A Systematic Review of the Literature. World Neurosurgery.

[3] Texakalidis Pavlos, Tzoumas Andreas, Xenos Dimitrios, Rivet Dennis J., Reavey-Cantwell John (2021). Carotid cavernous fistula (CCF) treatment approaches: A systematic literature review and meta-analysis of transarterial and transvenous embolization for direct and indirect CCFs. Clinical Neurology and Neurosurgery.

[4] Sun Peng, Chai Yuan, Fang Wei, Chen Hu, Long Qianfa, Zhao Zhenwei, Zhang Tao (2022). Case report: Spontaneous carotid-cavernous fistula associated with persistent primitive trigeminal artery aneurysm rupture. Frontiers in Neurology.

[5] Jiang Hui, Zeng Qun, Jiang Weimin (2023). Carotid-cavernous sinus fistula with primary clinical manifestation of cerebral infarction: description of two cases. Quantitative Imaging in Medicine and Surgery.

[6] Hamedani Hooman, Hellmann Daniel, Boyce William, Alesio Nicholas D’ (2022). Traumatic carotid-cavernous fistula: A case report. Radiology Case Reports.

[7] Krothapalli Neeharika, Fayad Mohamad, Sussman Eric, Bruno Charles, Ollenschleger Martin, Mehta Tapan (2023). Carotid cavernous fistula. Brain Circulation.

[8] Al-Boqami Beshayer A, Tammar Rahaf S, Alharbi Sultan E, Ahmed Zahraa M, Alharbi Ahlam (2023). Endovascular Intervention for a Carotid-Cavernous Fistula: A Case Report. Cureus.

[9] Sharma Rohan, Ponder Christian, Kamran Mudassar, Chacko Joseph, Kapoor Nidhi, Mylavarapu Krishna, Onteddu Sanjeeva, Nalleballe Krishna (2022). Bilateral Carotid-Cavernous Fistula: A Diagnostic and Therapeutic Challenge. Journal of Investigative Medicine High Impact Case Reports.

